# Supplementary material for: TET2 Mutation May Be More Valuable in Predicting Thrombosis in ET Patients Compared to PV Patients: A Preliminary Report
Source: J Clin Med. 2022 Nov 8;11(22):6615. doi: 10.3390/jcm11226615 (PMC9699342; doi:10.3390/jcm11226615)
Supplement: Supplementary file 1 [file jcm-11-06615-s001.zip › jcm-1986885-supplementary.pdf]

Supplementary Table S1. Specific sites and complications of thrombotic events in MPN patients with TET2 mutation.

No.	Disease	Gender	Age* (year)	Other gene mutation	Thrombosis	Infarction site	Complications
1	ET	Female	57	JAK2V617F	Acute coronary syndrome	The left anterior descending branch lumen stenosis was about 80%, and percutaneous coronary balloon dilatation angioplasty + stent placement was performed.	The postoperative recovery was good.
2	ET	Female	45	JAK2V617F	Ischemic stroke	The patient presented with right-sided limb numbness and cranial MRI showed cerebral infarction in the left basal ganglia region.	After treatment, there were no significant sequelae.
3	ET	Female	37	JAK2V617F	None		
4	ET	Female	50	JAK2V617F	Pulmonary embolism	Bilateral pulmonary artery embolism.	The patient improved with symptomatic treatment and is now recovering well.
5	ET	Male	67	JAK2V617F	Acute coronary syndrome	The posterior descending branch of the right coronary artery was 100% occluded, and percutaneous coronary balloon dilatation angioplasty + stent placement.	The postoperative recovery was good.
6	ET	Male	66	JAK2V617F	Acute coronary syndrome	Left middle anterior descending branch was 100% occluded, and percutaneous coronary balloon dilatation angioplasty + stent placement.	The postoperative recovery was good.
7	ET	Female	69	CALR	None		
8	ET	Male	50	CALR	None		
9	ET	Female	70	ASXL1	None		
10	PV	Male	61	JAK2V617F	Ischemic stroke	The patient presented to the clinic with unfavorable left limb movement with numbness, and the cranial MRI showed cerebral infarction with softening foci in the right hemi-oval central area-radiated coronal area.	he patient was left with weakness in the left limb.
11	PV	Male	70	JAK2V617F	None		

12	PV	Male	49	JAK2V617F	Ischemic stroke	The patient presented with sudden memory loss, and a cranial MRI showed a new cerebral infarction in the left basal ganglia region.	After treatment, the patient's memory was the same as before.
13	PV	Male	38	-	Ischemic stroke	The patient presented with numbness in the head for more than ten days, and a cranial CT showed multiple lacunar infarct foci in the left radiocoronal region.	After treatment, there were no significant sequelae.
14	PV	Male	33	JAK2V617F	None		
15	PV	Female	66	JAK2V617F	Ischemic stroke	The patient presented with unfavorable right limb movement and cranial MRI showed a new cerebral infarction in the left basal ganglia region and a punctate chronic ischemic foci in the right midbrain cerebral peduncle and right hemi-oval center.	The patient was left with weakness in the right limb.

*Age at diagnosis.
